# Super low threshold plasmonic WGM lasing from an individual ZnO hexagonal microrod on an Au substrate for plasmon lasers

**DOI:** 10.1038/srep08776

**Published:** 2015-03-05

**Authors:** H. M. Dong, Y. H. Yang, G. W. Yang

**Affiliations:** 1 State Key Laboratory of Optoelectronic Materials and Technologies, Institute of Optoelectronic and Functional Composite Materials, Nanotechnology Research Center, School of Physics & Engineering, Sun Yat-sen University, Guangzhou 510275, Guangdong, P. R. China

## Abstract

We demonstrate an individual ZnO hexagonal microrod on the surface of an Au substrate which can become new sources for manufacturing miniature ZnO plasmon lasers by surface plasmon polariton coupling to whispering-gallery modes (WGMs). We also demonstrate that the rough surface of Au substrates can acquire a more satisfied enhancement of ZnO emission if the surface geometry of Au substrates is appropriate. Furthermore, we achieve high Q factor and super low threshold plasmonic WGM lasing from an individual ZnO hexagonal microrod on the surface of the Au substrate, in which Q factor can reach 5790 and threshold is 0.45 KW/cm^2^ which is the lowest value reported to date for ZnO nanostructures lasing, at least 10 times smaller than that of ZnO at the nanometer. Electron transfer mechanisms are proposed to understand the physical origin of quenching and enhancement of ZnO emission on the surface of Au substrates. These investigations show that this novel coupling mode holds a great potential of ZnO hexagonal micro- and nanorods for data storage, bio-sensing, optical communications as well as all-optic integrated circuits.

Although surface plasmon-enhanced ultraviolet random lasing of ZnO films^1^ and nanowires^2^,^3^ have been achieved, they are still traditional photonic lasers. Optical confinement in traditional photonic lasers is restricted by the diffraction limit of the light which often demands large device sizes^4^,^5^. Very recently, many attentions have been paid to plasmon lasers^6^,^7^,^8^,^9^,^10^, which have been fabricated by CdS nanowires^11^ and nanosquares^12^ on the Ag substrate. Research showed that the geometry of metal substrates and dielectric layers has a crucial influence on the performance of plasmon lasers^13^,^14^,^15^. Additionally, surface plasmon is proposed to scale down their wavelength for nanoscale photonics, but the optical modes propagation loss increase rapidly on scaling the optical mode down^16^,^17^,^18^. Well known, ZnO nanostructures have attracted considerable interest due to great potential applications in micro- and nanoscaled optoelectronic devices, thus, understanding of a ZnO nanostructure such as a nanorod on the surface of a metal substrate has been becoming interesting for developing ZnO-based plasmon lasers. However, there have not been any reports involved in the system of a ZnO nanostructure on the surface of metal substrates for plasmon lasers, so far. On the other hand, micro- and nanoscaled whispering-gallery resonators with total internal reflection have the great potential for the applications of nanolasers and other nanophotonic devices^19^. ZnO hexagonal micro- and nanorods are regarded as important building blocks for nanoscaled optoelectronic devices, because they provide high quality (Q) factors leading to strong optical feedback within the micro- and nanocavity^20^,^21^,^22^,^23^,^24^,^25^. Therefore, both plasmon laser^12^ and whispering-gallery microcavity^26^ perform potential applications in ultrasmall-mode-volume devices.

In this contribution, we report a systemic study of an individual ZnO hexagonal microrod on the surface of Au substrates and we demonstrate that the surface plasmonic modes can strongly couple with the WGMs of ZnO hexagonal microrods to enhance the photoluminescence and split the WGMs on the rough surface of Au substrates. Importantly, we achieve high Q factor and super low threshold plasmonic WGM lasing in the fabricated systems, in which the Q factor is up to 5790 and the threshold is down to less than 0.5 KW/cm^2^ which is the lowest threshold value reported to date for ZnO nanostructures lasing, at least 10 times smaller than that of the most of ZnO at the nanoscale. Finally, we propose the physical understanding to the enhancement and quenching of the photoluminescence of ZnO on the surface of Au substrates. Therefore, these results are useful for design ZnO-based plasmon lasers. Note that many reports were involved in the case of ZnO nanowires covered by Au nanoparticles^27^,^28^,^29^,^30^,^31^. However, we suggest that the system of a ZnO nanostructure on the surface of Au substrates should be distinctly different from the case of ZnO nanowires covered by Au nanoparticles for plasmon lasers.

## Results

**Figure 1:**
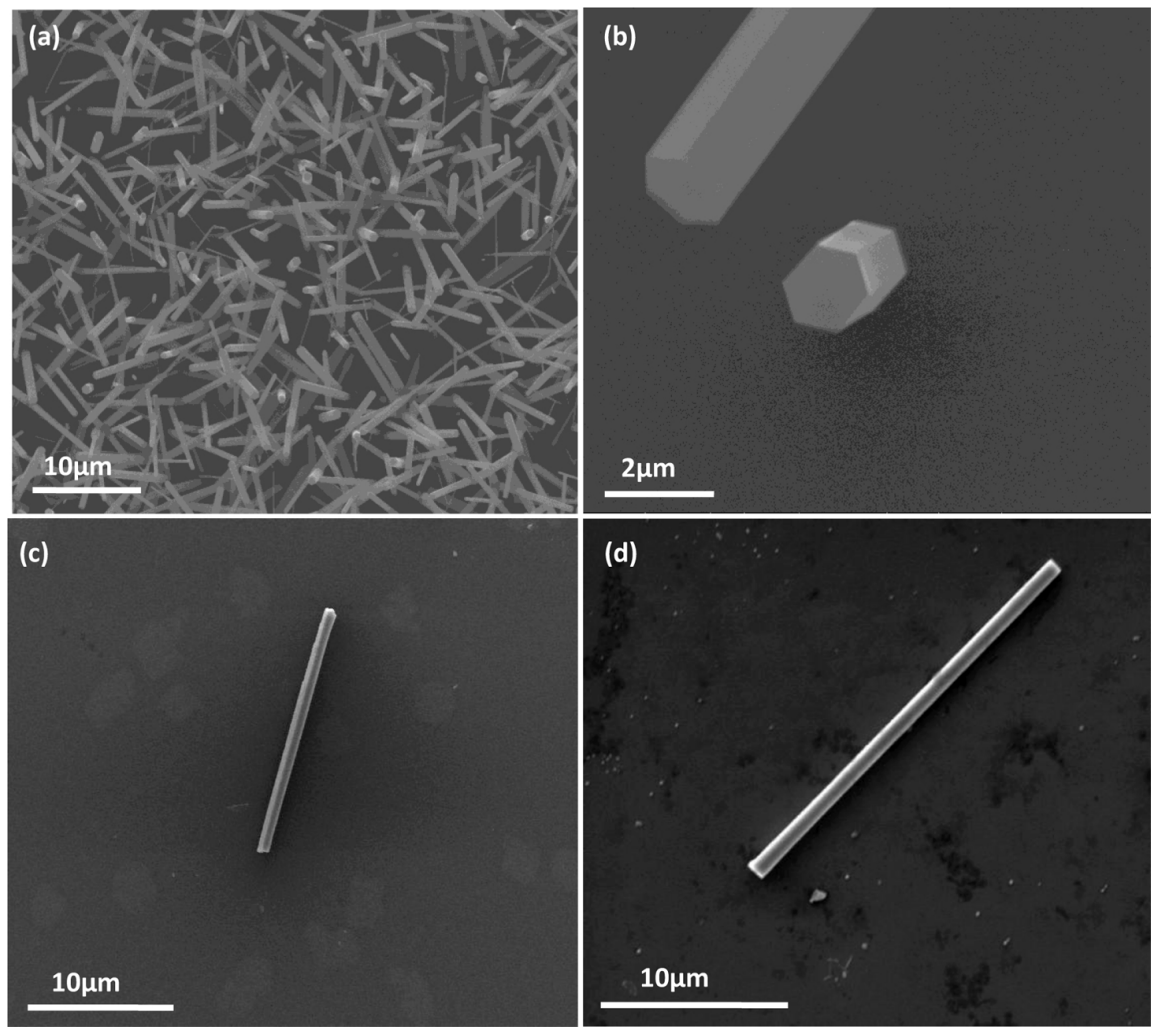
(a) and (b) The typical SEM images of the prepared ZnO hexagonal microrods in different magnifications. An individual ZnO microrod on the silicon substrate (c) and on the Au substrate (d).

**Figure 2:**
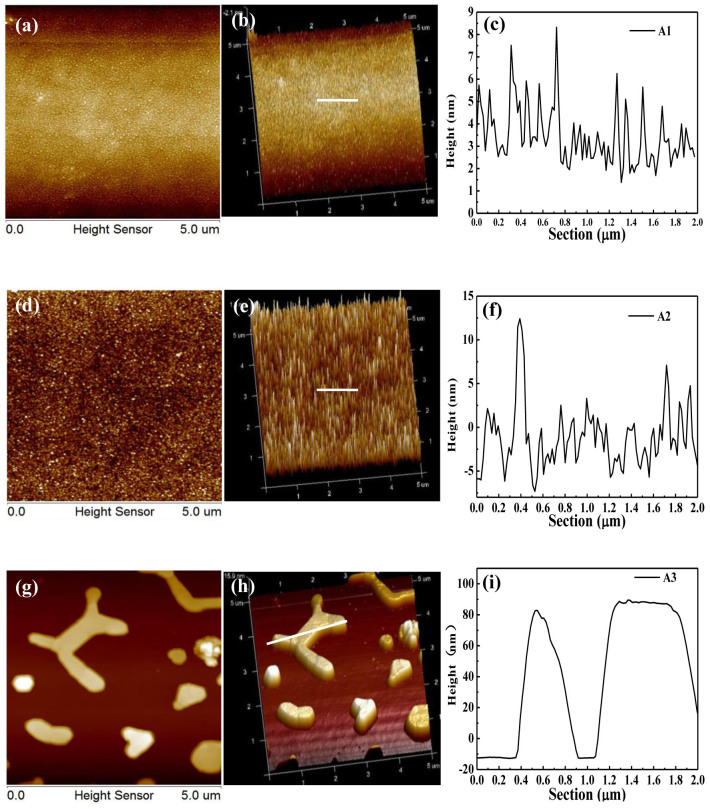
(a, d, g) 2D AFM images of the surface of A1, A2 and A3 substrates, respectively, (b, e, h) the corresponding AFM 3D images, and (c, f, i) the height of the section marked by a white light in 3D images. The scan size is 5 μm × 5 μm.

Figs. 1(a–b) show the typical SEM images of the prepared ZnO hexagonal microrods in different magnification. Clearly, these rods have perfect hexagonal shape with the size about 1–2 μm diagonal and 20 μm length, and their size is uniform. Fig. 1(c–d) show an individual ZnO microrod lies on silicon and Au substrates. Fig. 2 shows the different surface roughness of four Au substrates. We can clearly see that the surfaces of A1 and A2 substrates are rough, and the roughness increases with the thickness of gold films. However, the surface of the A3 substrate is the roughest among these substrates, because the surface actually consists of isolated nanoparticles.

**Figure 3:**
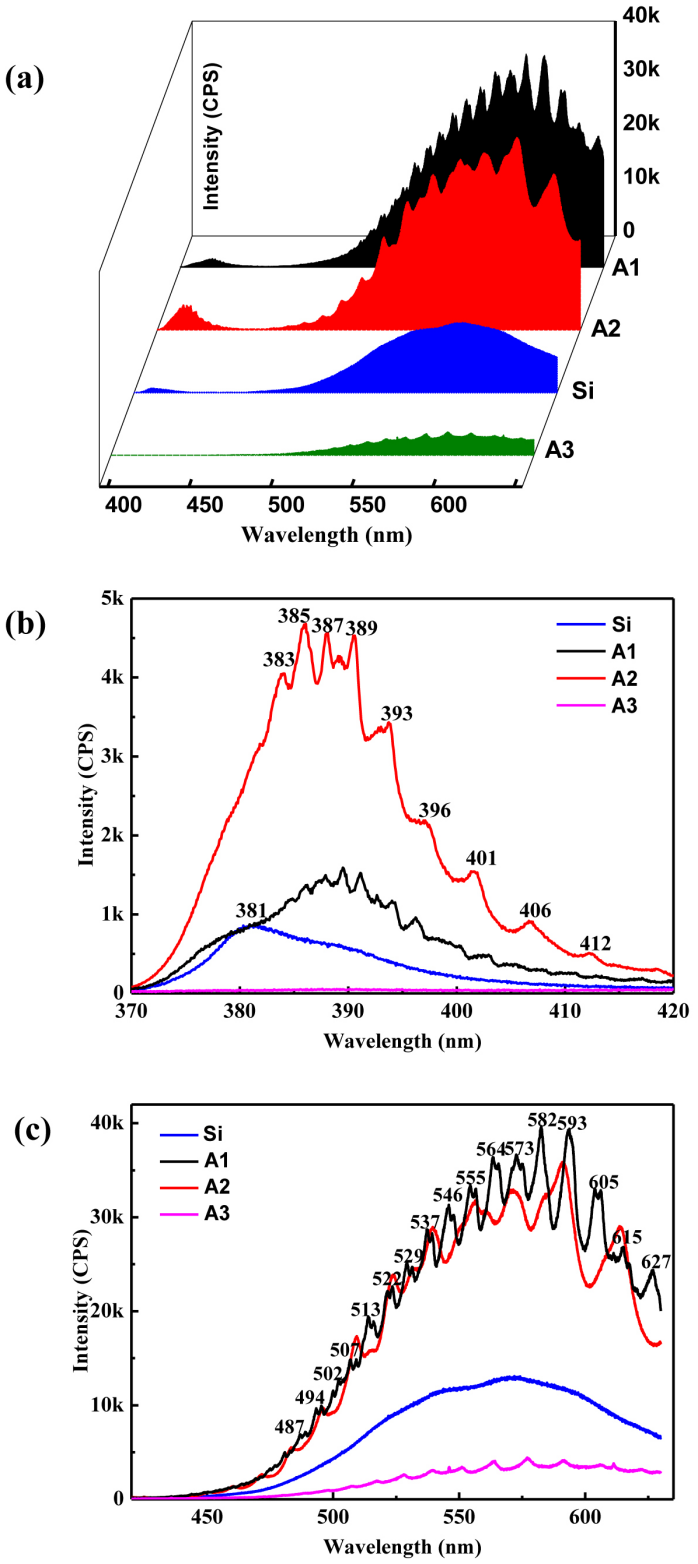
(a) PL spectra of an individual ZnO microrod on Si, A1, A2 and A3 substrates, respectively, in 3D waterfall. (b) The ultraviolet and (c) the visible emissions. Note that the mode number of WGM of ZnO is calculated in (b–c).

From Fig. 3, we can see that the different PL of ZnO microrods on Si, A1, A2, and A3 substrates, respectively. In detail, we can clearly see that the ultraviolet (UV) and defect emissions of ZnO are enhanced on A1 and A2 substrates compared with that of ZnO on the Si substrate, i.e., the rough surface of Au substrates can strongly enhance both UV and defect emissions of ZnO on the A1 and A2 substrates compared with that of the Si substrate in Fig. 3(a). Meanwhile, more WGMs of ZnO emission emerge on these substrates in Fig. 3(b–c). Therefore, these experimental observations show that the interaction between ZnO rod and substrate can greatly enhanced the WGMs of ZnO emission on the surface of Au substrates. In addition, there is a red shift both in UV and visible emissions of ZnO on these substrates. However, both UV and defect emissions of ZnO are quenching on the A3 substrate in Fig. 3(a). Thus, these results show that the enhancement of ZnO emission greatly depends on the surface roughness of Au substrates.

**Figure 4:**
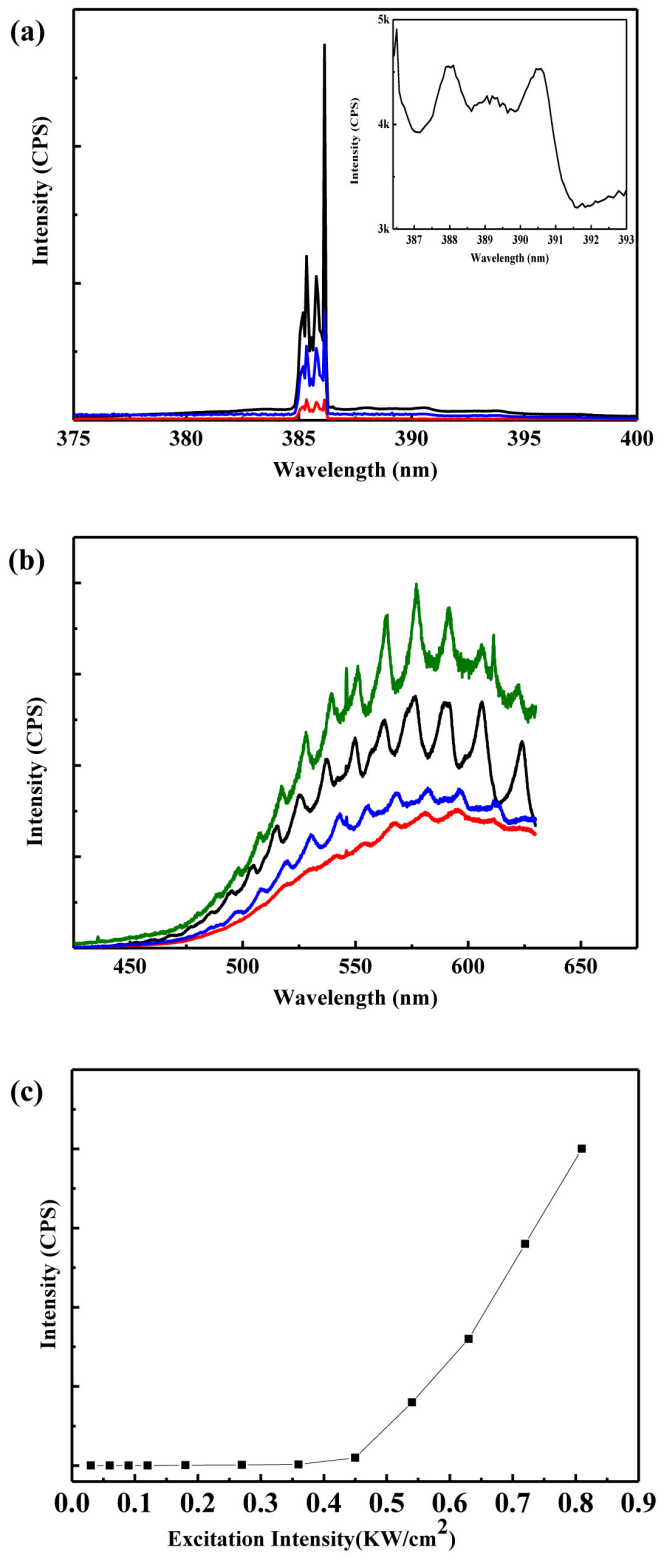
(a) The UV lasing spectra of a ZnO hexagonal rod on the A2 substrate and the excitation intensity is 0.38, 0.43, 0.56 KW/cm^2^, respectively. The inset is the enlarged spectrum between 386–390 nm. (b) The defect lasing data of ZnO hexagonal rod on A2 substrate. (c) Plot of emission peak intensity versus excitation intensity.

Further, taking an individual ZnO hexagonal microrod on the surface of the A2 substrate as an example, we study the lasing behavior and the results are shown in Fig. 4(a–c). Note that, in our case, for a ZnO rod on the bare Si substrate, no lasing on the WGM cavity modes is observed. From Fig. 4(a), we can see a strong UV lasing at 386 nm. Spectrum between 386–390 nm was enlarged in the inset of Fig. 4(a). It is clear that the mode was not cut off at 386 nm. It is just because the lasing peak is so high that makes other modes suppressed. The threshold value of lasing is an important parameter for the performance of devices. Fig. 4(b) shows the threshold values of an individual ZnO hexagonal microrod on the Au substrate by increasing the pumping energy. In detail, the lasing threshold value is typically found to be 0.45 KW/cm^2^. Remarkably, the threshold is down to less than 0.5 KW/cm^2^, which is the lowest threshold value reported to date for ZnO micro- and nanostructures lasing^39^, at least 10 times smaller than that of ZnO at the nanoscale. Fig. 4(c) shows the defect lasing experiment. No good lasing peak was observed, though it seems that two lasing mode at 546 nm and 612 nm may arise. By increasing the pumping energy, only the mode outline became clear.

For optical resonators, analyzing the observed resonance to understand the nature of the light confinement is very important. Fig. 3 shows obvious cavity modes despite being below the threshold, which indicates the excellent cavity feedback. The Q factor of ZnO microrods is given by *Q* = *λ*/Δ*λ*, where *λ* and Δ*λ* are the peak wavelength and its full width half maximum (FWHM). As there are many modes, we choose the strongest peak to calculate the Q factor under threshold. In the lasing condition above threshold, the Q factor of the A2 substrate is up to 5790 at the wavelength of 386 nm. Without appropriate cavity feedback, lasing phenomena could not happen. Various feedback mechanisms may account for the lasing, such as an F-P cavity, a random resonant effect or WGM. Since the lasing in our experiment is observed from a single ZnO disk, it cannot be attributed to random lasing caused by multiple scatting in a disordered medium. It is concluded in Ref. 32 that an F-P cavity provides a very low Q factor, as low as 468, which is much lower than our Q factor. It is also notable that the WGM is more preferable because of a better optical confinement. So we ascribe the observed lasing to WGM. These results suggest that the system of an individual ZnO hexagonal micro- and nanorod on the surface of Au substrates is expected to be a promising candidate for achieving ZnO-based plasmon laser.

Based on our experiment results, this hybrid system can really couple surface plasmon modes with WGMs, which can induce to a high Q factor below threshold. The Q factor of surface plasmon polaritons (SPPs) guided WGMs, *Q_SPP_*_+*WGM*_, is limited by two factors expressed as follow^27^
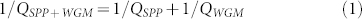
*Q_SPP_* is the metal loss experienced by the propagating SPP mode due to penetration into the gold layer. *Q_WGM_* is the radiation loss experienced by WGM. Therefore, these plasmonic WGMs bridge the space between traditional Fabry-Pero modes, WGMs and localized void plasmons. Such coupling of SPP and WGM works when the surrounding gold film provides the delocalized plasmon^28^. These results thus provide the important information that a ZnO hexagonal microrod with total internal reflection on the surface of Au substrates has a potential for plasmon laser if the surface geometry of Au substrates is appropriate. Furthermore, the clear signature of multiple cavity mode resonances at low pump powers demonstrates sufficient material gain to achieve full laser oscillation. Meanwhile the cavity feedback is abundant.

## Discussion

**Figure 5:**
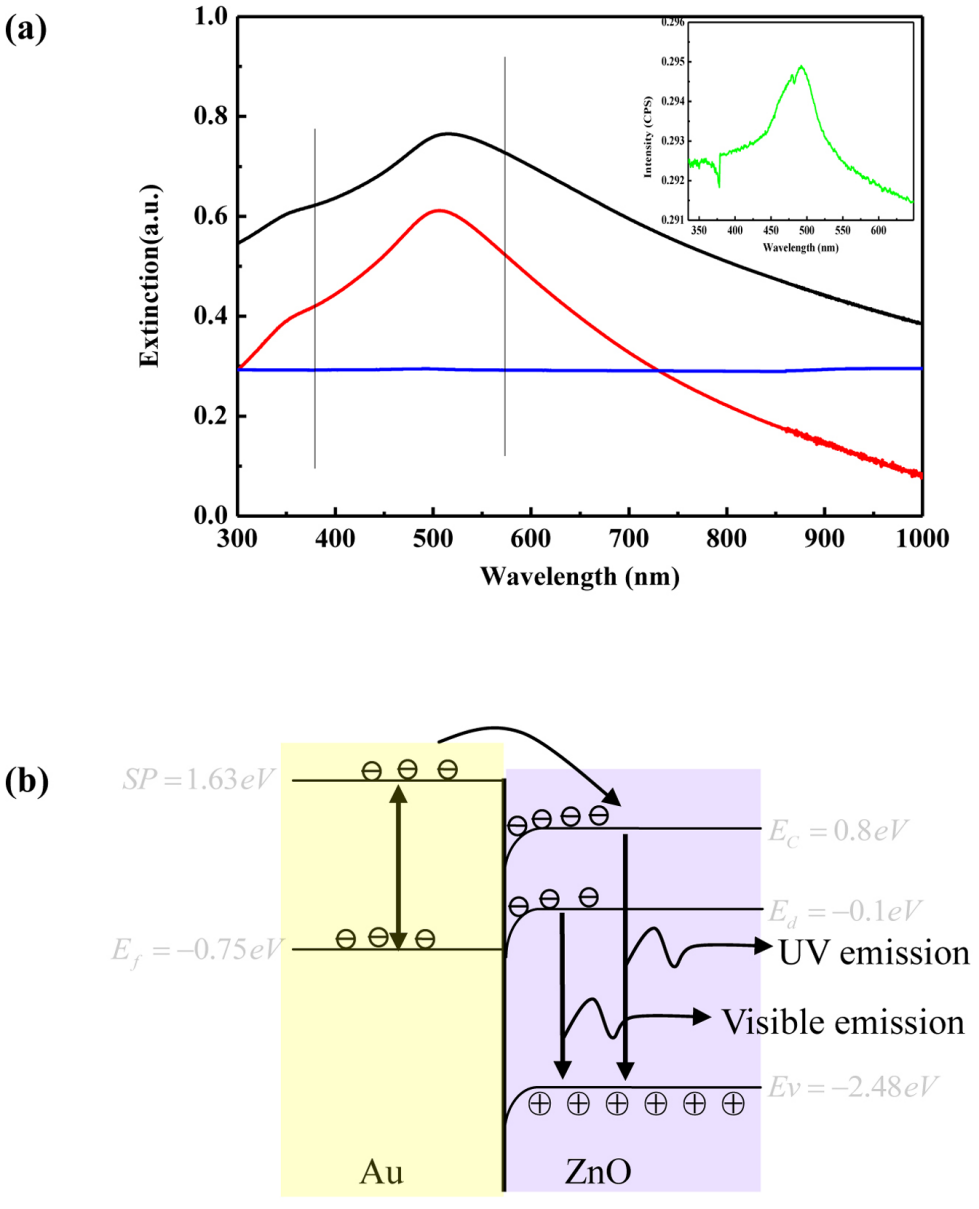
(a) Extinction spectra of A1, A2 and A3, respectively. The vertical line highlights the spectra position of emission bands of ZnO. A1 (red), A2 (black), A3 (blue). (b)Schematic diagrams of the electron transfer process of ZnO hexagonal microrod on the surface of the Au substrate.

For the ZnO micro- and nanorods, when excited by photons, electrons in the valence band (VB) can be excited to the conduction band (CB) creating electron-hole pairs. However, metal contacted with ZnO surface can alter its emission behavior through different mechanisms. Using the radiating surface plasmon mechanism, Lakowicz^31^ explain that surface plasmon resonance scattering of metal can enhance PL emission of semiconductors, while surface plasmon absorption can cause a quenching. In detail, PL enhancement in semiconductors usually occurs on rough metal surface or metal nanoparticles with large sizes, and quenching occurs on smoother metal surface or metal nanoparticles with much larger sizes. In order to gain the origin of enhancement and quenching in our experiments, the extinction spectra of the three Au prepared under different condition had been measured. It determines both absorption and scattering of the Au. Two SPR modes associated with the two axes of Au oblate spheroids were observed. The high energy band near 360 nm (out-of-plane mode) is due to the oscillation of the normal mode^33^, and the low energy band comes from the dipole oscillation parallel to the substrate plane (in-plane mode). As indicated in Fig. 5(a), the emission of ZnO at about 380 nm overlaps the longed wavelength spectral component of the out-of-plane resonance band of Au. It is known that due to the presence of the Au with proper rough and dipole-dipole interaction between the neighboring particles in the film, the SP band broadens in the longer wavelength spectral region and the scattering coefficient is raised greatly in that region^34^. Thus, by means of the effective SP radiative scatting the enhancement of the band gap emission at 380 is expected for A1 and A2. However, when the Au particles became too big and separated far from each other, the attenuation effect due to the reflection and absorption would dominate^35^. The inset in Fig. 5(a) which is the enlarged extinction spectrum of A3 shows that UV emission of A3 would not increase. For the defect emission at 560 nm, it located much further from the spectral component of the in-plane resonance band for A3. Under these conditions, the SP absorption attenuation is more effective than the radiative scattering, leading to the quenching of emission near 560 nm. However, the in-plane resonance band redshifts in A1 and A2. Consequently, the defect emission at 560 nm now approaches the wavelength spectral region of the in- plane resonance band. Then, the SP scatting at 560 nm will be raised and the emission can be effectively enhanced. It is suggested that only by proper resonance scattering of SP, the light emission can be enhanced.

In fact, electron transfer between semiconductor and metal takes place when they are in direct contact, and the direction of electron transfer depends on the band structures of semiconductors and the energy states of metal. To obtain a clear picture of the quenching and enhancement of PL in the system of ZnO hexagonal rods on the surface of Au substrates, the underlying mechanism is proposed as shown in Fig. 5(b). In drawing the band alignment, we use the data as follows. The conduction band of ZnO is located at −0.8 *eV* and the Fermi level of gold is −0.75 *eV* vs normal hydrogen electrode (NHE)^36^. As the large work function of ZnO, its Fermi energy level lies lower than the Femi energy level of gold, and the initial electron transfer between Au and ZnO will cause a band bending.

As the average particle size of the Au films deposited by sputtering is 4–100 nm, scattering dominates over absorption process. Electrons of Au films are excited to the surface plasmon level which is higher than the CB edge of ZnO, so electrons transfer to CB. The electrons accumulating in the potential well at the interface will increase substantially. Even if the photon generated by electron-hole pairs are separated at the interface, the radiactive recombination probability of electrons on CB with holes on VB will raise a lot due to the replenishment of electrons from Au, which results in an enhancement of the ultraviolet emission of ZnO. For the defect emission, since the Fermi level of Au is higher than the deep defect level of ZnO, it is therefore possible that the electrons can transfer from the Fermi level of Au to the deep defect level of ZnO. Thus the defect emission is enhanced^37^. And the wavelength of the plasmon is close to the defect wavelength. Thus, coupling of these two waves make it possible for PL enhancement. When the average size of the Au film becomes larger after being annealed, PL is quenching. Note that too much rough surface of Au film acts as an electron trap^38^. Electrons transfer from ZnO to Au, as a result, the defect emission is effectively suppressed and no enhancement for UV was observed. When the energy of surface plasmon is lower than that of excitons, energy transfers from excitons to surface plasmon occurs, which results in a red shift of the emission peaks of all.

In summary, we have demonstrated that the hybrid optical waveguide that consists of an individual ZnO hexagonal microrod on the surface of Au substrates suggests new sources that may manufacture miniature plasmon laser of ZnO by surface plasmon polariton coupling to WGMs. The lasing experiments of the fabricated ZnO hexagonal rod-Au substrates showed that the surface plasmon modes coupling with the WGMs can lead to a large Q factor and an abnormally low threshold for lasing, e.g., the Q factor is up to 5790 and the threshold is down to less than 0.5 KW/cm^2^ which is the lowest value reported to date for ZnO nanostructures lasing, at least 10 times smaller than that of ZnO at the nanometer. The physical understanding was proposed to address enhancement of ZnO emission on different surface of Au substrates based on electron transfer mechanisms. These findings make this unique coupling mode promising for a variety of applications in data storage, bio-sensing, optical communications as well as all-optic integrated circuits.

## Methods

### Materials preparation

The preparation of ZnO hexagonal microrods is conducted in a horizontal tube furnace by chemical vapor deposition. A mixture of commercial ZnO and graphite powders with a weight ratio of 1:1 is loaded on a quartz boat and posited at the higher temperature of the tube. Single-crystal Si wafers are cleaned by a standard procedure and placed downstream 3 cm far from the powder source at the lower temperature to act as a deposition substrate. Then, the system is heated to 1100°C for 60 min and kept at this temperature for 30 minutes without any catalyst in the air. After it was cooled down to room temperature, we observe a gray film on the substrate when it was moved out. A little and sharp knife is used to scrape the gray film into the deionized water and sonicating it for 2 minutes. Finally, the suspension is pipetted onto the Au substrates.

Au substrates are fabricated by depositing Au films on Si wafers. Two Au coatings with different thickness are deposited on Si wafers by a sputtering technique. The thickness of Au films is controlled by a current of 40 mA with two deposition times of 60 and 180 s, respectively. These two Au substrates are named to be A1 and A2 substrates. Additionally, another A2 substrate is annealed at 600°C in the air for 30 min to changing the surface morphology, which is named A3. The thicknesses of gold films on A1 and A2 substrates are about 40 and 90 nm, respectively. And the sizes of the gold particles on the A3 substrate are about 200 nm. In order to make the ZnO microrods attach to the surface of Au substrates tightly, all the samples are annealed at 100°C for 10 min.

### Characterizations

The fabricated systems were characterized by scanning electron microscopy (SEM) and the photoluminescence (PL), and lasing measurement was carried out by Renishaw inVia Raman Microscope with 325 nm excitation wavelength and the acquirement range was from 370 to 640 nm. The resolution of the microscope is in the order of μm, so we can acquire the PL and lasing from the individual ZnO microrod. The excitation laser of the μ-PL system is focused to a spot of 10 μm in diameter. The excitation laser source is continuous-wave He-Cd laser. The original power of the laser is 30 mW and the power of the laser reached the sample is 9 mW. A variable neutral density filter is used to attenuate the power. All these equipments are connected to a computer and operated in a WiRE-Single scan measurement system. In this system, the percent of the measured power can be chosen. The maximum excitation is about 11.5 KW/cm^2^. Then 0.05%–9% of the maximum excitation was used to acquire the lasing spectrum. Atomic force microscope (AFM) was used to characterize the surface roughness of various Au substrates. All measurements were carried out at room temperature.

## Author Contributions

G.W.Y. designed the experiments; H.M.D. carried out the experiments; Y.H.Y. carried out data analysis; H.M.D. and G.W.Y. wrote the paper.

## References

[b1] Abiyasa A. P., Yu S. F., Lau S. P., Leong E. S. P. & Yang H. Y. Enhancement of Ultraviolet Lasing from Ag-Coated highly Disordered ZnO Films by Surface-Plasmon Resonance. Appl. Phys. Lett. 90, 231106 (2007).

[b2] Tang W. *et al.* Surface Plasmon Enhanced Ultraviolet Emission and Observation of Random Lasing from Self-Assembly Zn/ZnO Composite Nanowires. CrystEngComm. 13, 2336 (2011).

[b3] Wang C.-S., Lin H.-Y., Lin J.-M. & Chen Y.-F. Surface-Plasmon-Enhanced Ultraviolet Random Lasing from ZnO Nanowires Assisted by Pt Nanoparticles. Appl. Phys. Exp. 5, 062003 (2012).

[b4] Liao L. *et al.* High Speed Silicon Mach-Zehnder Modulator. Opt. Exp. 13, 3129–3135 (2005). 10.1364/opex.13.003129 19495211

[b5] Green W. M., Rooks M. J., Sekaric L. & Vlasov Y. A. Ultra-compact, Low RF Power, 10 Gb/s SiliconMach-Zehnder Modulator. Opt. Exp. 15, 17106–17113 (2007). 10.1364/oe.15.017106 19551003

[b6] Oulton R. F., Sorger V. J., Genov D. A., Pile D. F. P. & Zhang X. A Hybrid Plasmonic Waveguide for Subwavelength Confinement and Long-Range Propagation. Nat. Photon 2, 496–500 (2008).

[b7] Sorger V. J. & Zhang X. Physics. Spotlight on Plasmon Lasers. Science 333, 709–710 (2011). 21817040 10.1126/science.1204862

[b8] Sorger V. J. *et al.* Experimental Demonstration of Low-Loss Optical Waveguiding at Deep Sub-Wavelength scales. Nat. Comm. 2, 331 (2011).

[b9] Chu H.-S., Li E.-P., Bai P. & Hegde R. Optical Performance of Single-Mode Hybrid Dielectric-Loaded Plasmonic Waveguide-Based Components. Appl. Phys. Lett. 96, 221103 (2010).

[b10] Zhang X.-Y. *et al.* Numerical Analysis of Deep Sub-Wavelength Integrated Plasmonic Devices Based on Semiconductor-Insulator-Metal Strip Waveguides. Opt. Exp. 18, 18945–18959 (2010). 10.1364/OE.18.018945 20940788

[b11] Oulton R. F. *et al.* Plasmon Lasers at Deep Subwavelength Scale. Nature 461, 629–632 (2009). 19718019 10.1038/nature08364

[b12] Ma R. M., Oulton R. F., Sorger V. J., Bartal G. & Zhang X. Room-Temperature Sub-Diffraction-Limited Plasmon Laser by Total Internal Reflection. Nat. Mater. 10, 110–113 (2011). 21170028 10.1038/nmat2919

[b13] Sidiropoulos T. *et al.* Ultrafast ZnO nanowire lasers: nanoplasmonic acceleration of gain dynamics at the surface plasmon polariton frequency. Nat. Phys. 10.1364/CLEO_QELS.2014.FTh3K.5 (2014).

[b14] Lee J., Shim H. S., Lee M., Song J. K. & Lee D. Size-Controlled Electron Transfer and Photocatalytic Activity of ZnO–Au Nanoparticle Composites. J. Phys. Chem. Lett. 2, 2840–2845 (2011).

[b15] Ruiz Peralta Mde L., Pal U. & Zeferino R. S. Photoluminescence (PL) Quenching and Enhanced Photocatalytic Activity of Au-decorated ZnO Nanorods Fabricated through Microwave-Assisted Chemical Synthesis. ACS Appl. Mater. & Interfaces 4, 4807–4816 (2012). 22939243 10.1021/am301155u

[b16] Gramotnev D. K. & Bozhevolnyi S. I. Plasmonics Beyond the Diffraction Limit. Nat. Photon 4, 83–91 (2010).

[b17] Maier S. A., Kik P. G. & Atwater H. A. Observation of Coupled Plasmon-Polariton Modes in Au Nanoparticle Chain Waveguides of Different Lengths: Estimation of Waveguide Loss. Appl. Phys. Lett. 81, 1714–1716 (2002).

[b18] Krasavin A. V. & Zayats A. V. Silicon-based Plasmonic Waveguides. Opt. Exp. 18, 11791–11799 (2010). 10.1364/OE.18.011791 20589040

[b19] Ilchenko V. S. & Matsko A. B. Optical Resonators with Whispering-Gallery Modes-part II: Applications. Selected Topics in Quantum Electronics, IEEE Journal of 12, 15–32 (2006).

[b20] Yang Y. H., Dong J. W., Wang N. W. & Yang G. W. Whispering Gallery Mode Enhanced Luminescence from an Individual ZnO Micro- and Nanoscaled Optical Resonator. J. Appl. Phys. 109, 093511 (2011).

[b21] Yang Y. H. *et al.* ZnO Nanocone: Application in Fabrication of the Smallest Whispering Gallery Optical Resonator. Nanoscale 3, 592–597 (2011). 21079824 10.1039/c0nr00592d

[b22] Wang N. W. *et al.* General Strategy for Nanoscopic Light Source Fabrication. Adv. Mater. 23, 2937–2940 (2011). 21567480 10.1002/adma.201100508

[b23] Wang N. W. *et al.* Diffuse Reflection inside a Hexagonal Nanocavity. Sci. Rep. 3, 1298 (2013). 23416646 10.1038/srep01298 PMC3575014

[b24] Dong H., Yang Y. & Yang G. Directional Emission from ZnO Hexagonal Disks. ACS Appl. Mater. & Interfaces 6, 3093–3098 (2014). 24552159 10.1021/am4058869

[b25] Min B. *et al.* High-Q Surface-Plasmon-Polariton Whispering-Gallery Microcavity. Nature 457, 455–458 (2009). 19158793 10.1038/nature07627

[b26] Chen Y.-H. & Guo L. J. High Q Long-Range Surface Plasmon Polariton Modes in Sub-wavelength Metallic Microdisk Cavity. Plasmonics 6, 183–188 (2010).

[b27] Cole R. *et al.* Easily Coupled Whispering Gallery Plasmons in Dielectric Nanospheres Embedded in Gold Films. Phys. Rev. Lett. 97, 137401 (2006). 17026072 10.1103/PhysRevLett.97.137401

[b28] Hon N. K. & Poon A. W. In: Surface Plasmon Resonance Enhanced Coupling to Whispering-Gallery Modes in Optical Micropillar Resonators. Conference on Lasers and Electro-Optics/Quantum Electronics and Laser Science Conference and Photonic Applications Systems Technologies, Long Beach, California, Optical Society of America: Long Beach, California, p QMI2 (2006, 05).

[b29] Shopova S. I., Rajmangal R., Holler S. & Arnold S. Plasmonic Enhancement of a Whispering-Gallery-Mode Biosensor for Single Nanoparticle Detection. Appl. Phys. Lett. 98, 243104 (2011).

[b30] Chen Z. X. *et al.* Hybrid material based on plasmonic nanodisks decorated ZnO and its application on nanoscale lasers. Nanotechnology 25, 295203 (2014). 24990516 10.1088/0957-4484/25/29/295203

[b31] Lakowicz J. R. Radiative Decay Engineering 5: Metal-Enhanced Fluorescence and Plasmon Emission. Anal. Biochem. 337, 171–194 (2005). 15691498 10.1016/j.ab.2004.11.026 PMC2763912

[b32] Chen R., Ling B., Sun X. W. & Sun H. D. Room Temperature Excitonic Whispering Gallery Mode Lasing from High-Quality Hexagonal ZnO Microdisks. Adv. Mater. 23, 2199–2204 (2011). 21462376 10.1002/adma.201100423

[b33] Royer P., Goudonnet J., Warmack R. & Ferrell T. Substrate effects on surface-plasmon spectra in metal-island films. Phys. Rev. B 35, 3753–3759 (1987). 10.1103/physrevb.35.3753 9941895

[b34] Shinada S., Hashizume J. & Koyama F. Surface plasmon resonance on microaperture vertical-cavity surface-emitting laser with metal grating. Appl. Phys. Lett. 83, 836 (2003).

[b35] Giannattasio A., Hooper I. & Barnes W. Transmission of light through thin silver films via surface plasmon-polaritons. Opt. Express 12, 5881–5886 (2004). 19488227 10.1364/opex.12.005881

[b36] Mahanti M. & Basak D. Enhanced Emission Properties of Au/SiO_2_/ZnO Nanorod Layered Structure: Effect of SiO_2_ Spacer Layer and Role of Interfacial Charge Transfer. RSC Adv. 4, 15466 (2014).

[b37] Zhou X. D. *et al.* Mechanism of the enhancement and quenching of ZnO photoluminescence by ZnO-Ag coupling. EPL (Europhysics Letters) 93, 57009 (2005).

[b38] Zheng J., Zhou C., Yu M. & Liu J. Different Sized Luminescent Gold Nanoparticles. Nanoscale 4, 4073–4083 (2012). 22706895 10.1039/c2nr31192e PMC3404491

[b39] Choy J. H., Jang E. S. & Won J. H. *et al.* Hydrothermal route to ZnO nanocoral reefs and nanofibers. Appl. Phys. Lett. 84, 287–289 (2004).

